# When Coke Is Not Hydrating: Cocaine-Induced Acute Interstitial Nephritis

**DOI:** 10.1177/2324709614551557

**Published:** 2014-09-30

**Authors:** Mohammed Bahaa Aldeen, Nibras Talibmamury, Sumer Alalusi, Omar Nadham, Abdel Rahman Omer, Roger D. Smalligan

**Affiliations:** 1 Texas Tech University Health Sciences Center, Amarillo, TX, USA

**Keywords:** cocaine induced acute interstitial nephritis

## Abstract

A 47-year-old African American man was admitted with 4 days of back pain, nausea and vomiting, and low urine output. There was no history of fever, dysuria, frequency, hesitancy, viral symptoms, trauma, rash, or constipation. Despite his past medical history of hypertension, diabetes mellitus, and hyperlipidemia he denied taking any medications for 18 months, including nonsteroidal anti-inflammatory drugs, acetaminophen, or antacids. He denied smoking and alcohol but admitted to cocaine use. No significant FH. Physical examination results were as follows: BP 235/125 mm Hg, heart rate 90 beats/min, temperature 98°F, O_2_ saturation normal; lungs and heart normal, abdomen soft but bilateral costovertebral angle tenderness. Neurological examination was normal. Laboratory tests yielded the following results: creatinine (Cr) 10.5 mg/dL (1.2 mg/dL in 2010), blood urea nitrogen 63 mg/dL, glucose 151 mg/dL, Ca 9.4 mg/dL, PO_4_ 6.1 mg/dL, Hgb 15 g/dL, white blood cells (WBC) 9100, platelets 167 000, amylase/lipase normal, aspartate aminotransferase/alanine aminotransferase (AST/ALT) normal, bilirubin 1.4 mg/dL, alkaline phosphatase 39 IU/L, creatine phosphokinase 127 µg/L. Hepatic panel, C- and P-ANCA (cytoplasmic– and perinuclear–antineutrophil cytoplasm antibodies, respectively), anti-GBM (anti–glomerular basement membrane), antimyeloperoxidase, antinuclear antibody, and *Helicobacter pylori* were all negative. C3, C4 normal, urinalysis: 2+ blood, no white blood cells or eosinophils, no casts, no albumin, negative for nitrate/leukocyte esterase and bacteria. Imaging: chest radiograph, abdominal radiograph, computed tomography of the abdomen, electrocardiography, and transthoracic echocardiography were all normal. *Course*. The patient’s urine output declined from 700 to 400 cm^3^/d and the on third day he required hemodialysis with Cr 14 mg/dL. Renal biopsy showed typical findings of interstitial nephritis. The patient was dialyzed for 10 days and responded to steroids and went home with an improving Cr of 3.5 mg/dL, back to baseline of 1.5 in 8 weeks. *Discussion*. Internists encounter patients with acute kidney injury (AKI) on a daily basis, most of which can be explained by prerenal azotemia, acute tubular necrosis (ATN), obstruction, or rhabdomyolysis among other etiologies. Cocaine is only rarely implicated as an etiology of AKI and if it is, usually the injury is due to ATN or pigment effects. Acute interstitial nephritis (AIN) caused by cocaine, on the other hand, has only been described in a handful of cases. AIN is a renal lesion that causes a decline in creatinine clearance and is characterized by an inflammatory infiltrate in the kidney interstitium and is most often associated with drug therapy. AIN can also be seen in autoimmune disorders like systemic lupus erythematosus, Sjögren’s syndrome, or sarcoidosis; or with infections remote to the kidney like Legionella, leptospirosis, and streptococcal disease. Our case was very similar to the other reported cases of AIN due to cocaine in that all have occurred in middle-aged African American males and all have responded to steroids. This case reminds clinicians to consider AIN in patients with AKI and a history of cocaine abuse.

## Introduction

Acute interstitial nephritis (AIN) is a renal lesion that causes a decline in creatinine clearance and is characterized by an inflammatory infiltrate in the kidney interstitium.^1^ It is most often induced by drug therapy but is also seen in autoimmune disorders. In the era of increased drug abuse, we report an uncommon cause of interstitial nephritis: cocaine abuse.

## Case Report

A 47-year-old African American man came to the emergency department with complaints of 4 days of severe, colicky back pain with some radiation to the flanks, associated with nausea and vomiting and a noticeable decrease in urination. He denied any history of trauma, stones, dysuria, frequency, hesitancy, rash, constipation, diarrhea, fever, or previous similar pain. He denied any unusual food or sick contact. He denied taking any medications for the past 18 months, including nonsteroidal anti-inflammatory drugs (NSAIDs), acetaminophen, and antacids. He denied smoking, alcohol, and drugs although it had been noted in the chart that he had a positive urinary toxicology screen in the past for cocaine. Although he had a history of hypertension, diabetes, and hyperlipidemia diagnosed 3 years previously during a brief hospital stay, he was not taking any of the recommended prescription medications. Family history was positive for premature atherosclerosis, yet negative for autoimmune diseases and tuberculosis. On physical examination, his vital signs included blood pressure of 235/125 mm Hg, pulse 90 beats/min, respiratory rate 20/min, temperature 37°C, O_2_ saturation 96% on room air. He was awake and alert, had clear lungs, a regular heart rate with no murmurs, gallops, or rubs, a soft abdomen with normal bowels sounds, bilateral costophrenic tenderness to percussion, a normal prostate, and neurologic examination.

### Laboratory Tests

Creatinine was 10.48 mg/dL, up from his previous level of 1.15 mg/dL at his previous visit 3 years prior. Blood urea nitrogen 63 mg/dL, Na 134 mEq/L, K 4.4 mEq/L, Cl 100 mEq/L, CO_2_ 22 mEq/L, glucose 151 mg/dL, Ca 9.4 mg/dL, PO_4_ 6.1 mg/dL, Mg 2.1 mg/dL, hemoglobin 15 g/dL, hematocrit 45%, white blood cells (WBC) of 9100 with neutrophils 75%, lymphocytes 12.5%, and eosinophils 0.9%, platelets of 167 000. His troponin was 0.02 ng/mL, CK-MB 2.6 ng/mL, BNP 53 pg/mL, amylase 70 U/L, lipase 69 U/L, international normalized ratio 1.1, prothrombin time 13 seconds, partial thromboplastin time 31 seconds, HbA1c 6.6%, aspartate transaminotransferase (AST) 25 IU/L, alanine aminotransferase (ALT) 20 IU/L, albumin 3.8 g/dL, total protein 7.3 g/dL, bilirubin 1.4 mg/dL, alkaline phosphatase 39 IU/L, C-reactive protein 25 mg/L. Urine toxicology screen was positive for cocaine. Urinalysis showed 2+ blood, no red blood cells, no WBCs, including no eosinophils by special stain, no casts, no albumin, negative for nitrates, negative leukocyte esterase and negative for bacteria, trace ketone, pH 5.5, and specific gravity 1.015. No pigmented granular casts. His hepatitis panel: C-ANCA (cytoplasmic–antineutrophil cytoplasm antibodies) Ag, P-ANCA (perinuclear–antineutrophil cytoplasm antibodies), anti–glomerular basement membrane Ag, anti-myeloperoxidase, antinuclear antibody and *Helicobacter pylori* IgA were all negative. Creatine phosphokinase was 127 mg/dL and his serum electrophoresis was normal. C3 was 119 mg/dL and C4 was 88.5 mg/dL (both normal). Anti-proteinase 3 (PR-3) antibodies were positive.

### Imaging and Electrocardiography

His chest and abdominal radiographs were normal, and computed tomography of the abdomen and pelvis without contrast showed no urinary obstruction. Transthoracic echocardiogram showed normal wall motion and ejection fraction. Electrocardiography showed nonspecific T-wave abnormalities, which were unchanged since 2010.

### Hospital Course

The patient’s blood pressure was initially controlled with intravenous labetalol and hydralazine. Over the first 3 days of admission, his urinary output decreased from 700 cm^3^/d to less than 400 cm^3^/d and his creatinine rose to 13.6 mg/dL. Nephrology was consulted early and after ruling out acute toxic nephropathy, urinary obstruction, and acute rhabdomyolsis, it was suspected, based on his initial positive toxicology screen for cocaine that he may have cocaine-induced interstitial nephritis and a renal biopsy was performed (see below). Indeed, on further questioning, the patient admitted to smoking crack cocaine. It was confirmed with the patient and his spouse that the cocaine was not mixed or combined with any other substance. At that point (day 4) the patient was started on intravenous methylprednisolone at 125 mg every 6 hours as well as daily or every other day hemodialysis for a total of 6 sessions. The methylprednisolone was tapered to 80 mg every 6 hours after 3 days and then changed to oral prednisone, which was furthered tapered over the next 12 days. The steroid was used roughly day 4 of admission, and 7 days after symptoms had started, it is hard to tag a response to the steroid separately as the patient was started on both hemodialysis and steroid at the same time after biopsy of kidney confirmed the diagnosis; nevertheless, we were able to see stable improvement on creatinine on days 8 and 9, with improvement of urine output (roughly 1000-1500 cm^3^/d). The patient stabilized with his creatinine at 3.54 mg/dL before being discharged home and follow-up 8 weeks later showed a normal creatinine.

### Renal Biopsy

**Figure 1.**
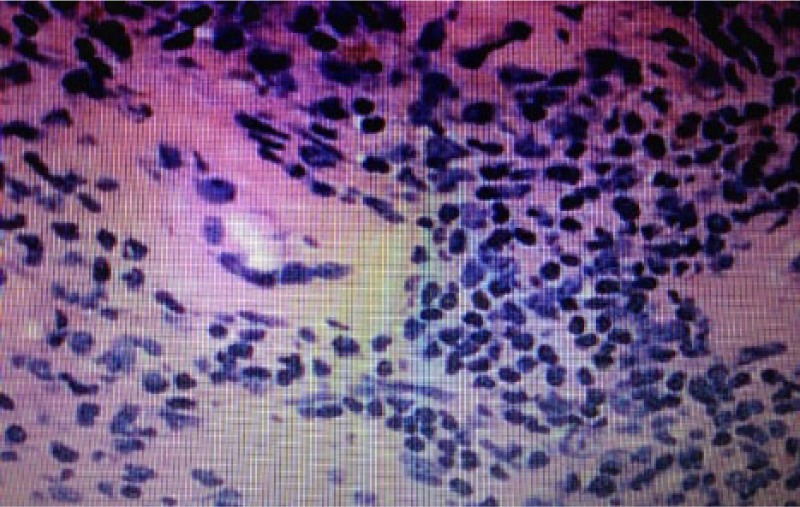
Renal biopsy showing interstitial inflammation with lymphocytes, plasma cells, eosinophils, and edema with areas of arterioarterosclerosis consistent with interstitial nephritis.

Renal biopsy (Figure 1) showed normocellular glomeruli, interstitial atrophy, and fibrosis with tubular loss of 10% to 20%. There were foci of interstitial inflammation composed of lymphocytes, plasma cells, eosinophils, and edema with areas of severe arterioarterosclerosis. No immune complexes were detected.

## Literature Review

In addition to our case, a PubMed search revealed 4 other cases of cocaine-induced AIN. Table 1 compares the findings in the other 4 cases with our case.

**Table 1.** Clinical Features of Cocaine-Induced Acute Interstitial Nephritis.

Authors (Year)	Alvarez et al (1999)^2^	Decelle et al (2007)^3^	Wojciechowski et al (2008)^4^	Alfaro et al (2013)^5^	Our Case (2013)
Drug	Cocaine	Cocaine	Cocaine	Cocaine	Cocaine
Sex	Male	Male	Male	Male	Male
Age (year)	34	42	38	49	47
Abdominal pain	Yes	Yes	Yes	Yes	Yes
Oliguria	Yes	Yes	Yes	No	Yes
Hematuria	Yes	Yes	Yes	Yes	Yes
Proteinuria	Yes	Yes	Yes	Yes	No
Eosinophiluria	No	No	Yes	No	No
Blood urea nitrogen (mg/dL)	113	332	91	84	63
Creatinine (mg/dL)	Admission: 1.0 Day 6: 12.5	Admission: 20.5 Day 9: 1.7	13	12.8	Admission: 10.89 Day 3: 14.26
Dialysis	Yes	Yes	Yes	Yes	Yes
Steroid	No	Yes (intravenous)	Yes (intravenous)	No	Yes (intravenous + oral)
Recovery kidney function	Yes	Yes	Yes	Yes	Yes

## Discussion

Physicians encounter patients with acute kidney injury on a daily basis, most of which can be explained by prerenal azotemia, acute tubular necrosis, obstruction, or rhabdomylosis among other etiologies. Cocaine is only rarely implicated as an etiology of acute kidney injury and if it is, usually the injury is due to acute tubular necrosis or pigment effects. As demonstrated in the literature review, AIN caused by cocaine has only been described in a handful of cases. The clinical presentation of AIN can range from almost asymptomatic oliguria to fever, rash, malaise, nausea, vomiting, and abdominal pain. A significant decline in creatinine clearance is consistent and renal biopsy will show an inflammatory infiltrate in the kidney interstitium as described above.^6^ The most common association is with drug therapy, including a long list of antibiotics, NSAIDs, diuretics, and other miscellaneous medications such as acyclovir, allopurinol, and aziathioprine. AIN can also be seen in autoimmune disorders such as systemic lupus erythematosis, Sjögren’s syndrome, and sarcoidosis; or with infections remote to the kidney such as Legionella, leptospirosis, and streptococcal disease.^6,7^

The mechanism of AIN likely involves an immunologic disturbance, possibly a delayed hypersensitivity reaction.^8^ Most often the offending agents are drugs. Evidence suggests that drug-induced AIN is secondary to immune reactions in humans since AIN occurs only in a small percentage of individuals taking the particular drugs and it is not dose dependent. Drug-induced AIN is associated with extrarenal manifestations of hypersensitivity and in fact it usually recurs after accidental reexposure to the drug or to a closely related agent.^8^ Definitive diagnosis may require renal biopsy, which will reveal normal glomeruli and patchy but usually heavy interstitial infiltration of lymphocytes, plasma cells, and eosinophils. Prompt and accurate diagnosis of AIN is important because withdrawal of the offending agent will usually result in rapid improvement in renal function and steroid therapy may reduce residual chronic renal damage.

Corticosteroid therapy has been employed to treat AIN that persists despite discontinuation of the offending agent. This is usually instituted three to seven days after lack of improvement is noted.^1,6,8,9^ However, the benefits of therapy are inconclusive since the available data are conflicting and there are no randomized controlled trials. There are several possible explanations for a lack of effect from glucocorticoids in the negative studies in the literature: The patients treated with steroids may have had more severe disease, as manifested by higher maximum serum creatinine concentrations in some studies, a significant proportion of the patients had NSAID-associated AIN, which is less likely to respond to glucocorticoid therapy.^6,10,11^ Given the potential for benefit and the relative safety of short-term therapy, it seems reasonable to treat patients with corticosteroids if they do not have significant improvement in the serum creatinine within 3 to 7 days after discontinuation of the offending agent.^8,9,12^

Although our patient was positive for PR3 antibodies, the patient did not have any skin or mucosal lesions, his urinalysis showed no casts, his chest radiograph showed no infiltrates and more important, his kidney biopsy clearly showed normal glomeruli with typical features of AIN. PR3 antibodies directed against c-ANCA are present in a number of conditions, including granulomatosis with polyangiitis (Wegener’s),^13^ microscopic polyangiitis, pauci-immune crescentic glomerulonephritis, Churg–Strauss syndrome, cystic fibrosis, inflammatory bowel disease, primary sclerosing cholangitis, and rheumatoid arthritis. The presence or absence of ANCA does not indicate definitively the presence or absence of any of these diseases as one must rely on clinical features as well. While our patient had PR3 antibodies, there are no data on how commonly PR3 coexists with AIN.

Our case was extremely similar to the other reported cases of AIN due to cocaine. All such reported cases have occurred in African American males, all have been in middle age, and all have responded well to steroids and temporary dialysis when necessary. This case reminds clinicians to consider AIN in patients with acute kidney injury and a history of cocaine abuse.
